# Enabling the design of surgical instruments for under-resourced patients through metal additive manufacturing: ulnar shortening osteotomy as an example

**DOI:** 10.1186/s41205-024-00220-3

**Published:** 2024-05-31

**Authors:** Kuan-Lin Chen, Cheng-Yu Yin, Hui-Kuang Huang, Yi-Chao Huang, Jung-Pan Wang

**Affiliations:** 1 Department of Orthopaedics & Traumatology, Taipei Veterans General Hospital, No.201, Sec 2, Shih-Pai Road, Taipei, Taiwan; 2 Division of Joint Reconstruction, Taipei Veterans General Hospital, Taipei, Taiwan; 3 Biomechanics & Tendon and Soft Tissue Biology Laboratory, Division of Orthopedic Research, Mayo Clinic, Rochester, USA; 4 Department of Orthopedic Surgery, School of Medicine, National Yang Ming Chiao Tung University, Taipei, Taiwan; 5 Department of Orthopaedics, Ditmanson Medical Foundation Chiayi Christian Hospital, Chiayi, Taiwan

**Keywords:** Metal additive manufacturing, Surgical instrument design, Ulnar shortening osteotomy

## Abstract

**Background:**

Ulnar shortening osteotomy (USO) has demonstrated good outcomes for patients with ulnar impaction syndrome. To minimize complications such as non-union, precise osteotomy and firm fixation are warranted. Despite various ulnar shortening systems have been developed, current technology does not meet all needs. A considerable portion of patients could not afford those designated USO systems. To tackle this challenge, our team reported successful results in standardized free-hand predrilled USO technique. However, it is still technical demanding and requires sufficient experience and confidence to excel. Therefore, our team designed an ulnar shortening system based on our free-hand technique principle, using metal additive manufacturing technology. The goal of this study is to describe the development process and report the performance of the system.

**Methods:**

Utilizing metal additive manufacturing technology, our team developed an ulnar shortening system that requires minimal exposure, facilitates precise cutting, and allows for the easy placement of a 3.5 mm dynamic compression plate, available to patients at zero out-of-pocket cost. For performance testing, two surgeons with different levels of experience in ulnar shortening procedures were included: one fellow-trained hand and wrist surgeon and one senior resident. They performed ulnar shortening osteotomy (USO) using both the free-hand technique and the USO system-assisted technique on ulna sawbones, repeating each method three times. The recorded parameters included time-to-complete-osteotomy, total procedure time, chip diameter, shortening length, maximum residual gap, and deviation angle.

**Results:**

For the hand and wrist fellow, with the USO system, the time-to-complete osteotomy was significantly reduced. (468.7 ± 63.6 to 260.0 ± 5 s, *p* < 0.05). Despite the preop goal was shortening 3 mm, the average shortening length was significantly larger in the free-hand group (5 ± 0.1; 3.2 ± 0.2 mm, *p* < 0.05). Both maximum residual gap and deviation angle reported no statistical difference between the two techniques for the hand surgeon. As for the senior resident, the maximum residual gap was significantly reduced, using the USO system (2.9 ± 0.8; 0.4 ± 0.4 mm, *p* = 0.02). Between two surgeons, significant larger maximum residual gap and deviation angle were noted on the senior resident doctor, in the free-hand technique group, but not in the USO system group.

**Conclusion:**

The developed USO system may serve as a valuable tool, aiding in reliable and precise cutting as well as fixation for patients undergoing ulnar shortening osteotomy with a 3.5 mm dynamic compression plate, even for less experienced surgeons. The entire process, from concept generation and sketching to creating the CAD file and final production, serves as a translatable reference for other surgical scenarios.

## Background

Ulnar impaction syndrome, caused by a positive ulnar variance is a common cause of ulnar-sided wrist pain. Ulnar shortening surgery has demonstrated good outcomes for patients with ulnar impaction syndrome [1]. Significant improvement of functional scores were reported [2]. Most common complications include delayed union and non-union [3–5]. To optimize the outcome of USO and minimize the possibility of non-union, a precise osteotomy and firm fixation is warranted. Therefore, various companies have developed ulnar shortening systems, providing an easy cutting and fixation workflow [6–8]. However, many patients can not afford the cost of those systems, and most of these USO-specific cutting systems can not be applied to traditional dynamic compression plates or regular locking plates, which poses lower out of pocket cost. Therefore, hand surgeons would complete the procedure using free-hand technique followed by fixation with a 3.5 mm dynamic compression plate or a 3.5 mm locking plate. However, free-hand technique is technical dependent. As a result, without sufficient experience, many surgeons tend to be conservative in bringing up USO as a treatment option for those who may benefit from the surgery. To flatten the learning curve of performing ulnar shortening osteotomy, based on the published “predrilled free-hand technique for USO”, leveraging metal additive manufacturing technology, our team developed an ulnar shortening osteotomy system for commercially available 3.5 mm dynamic compression plate to facilitate accurate osteotomy and implant fixation [9]. Additive manufacturing technology enabled hospital in-house surgical system design under small budget. This study hypothesizes that the additive-manufactured ulnar shortening osteotomy system can produce more accurate and repeatable results among surgeons with varying levels of experience compared with free-hand technique.

## Methods

### Designing the ulnar shortening system

The goal of this system is to facilitate precise osteotomy, reduction, and plate fixation using a 3.5 mm Dynamic Compression Plate (Synthes, Switzerland). The system includes two important components: a predrilled hole aiming guide and an osteotomy cutting jig.

The most challenging part of osteotomy is making a parallel cut at a designated thickness to achieve effective shortening and allow anatomical reduction. A skewed cut increases the risk of malunion or non-union. The cutting jig must be able to fix securely to the ulna to withstand the vibration from the oscillating saw during cutting. Inadequate fixation could lead to the loosening of the cutting jig, resulting in inaccurate cutting.

**Fig. 1:**
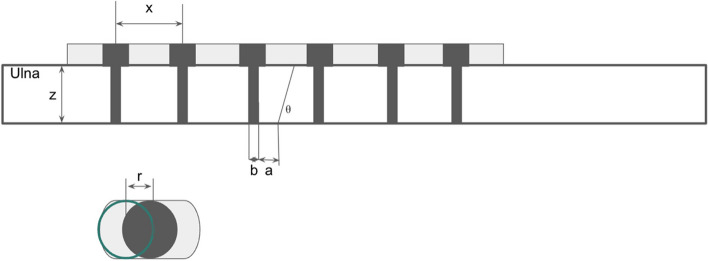
Illustration of the quantitative considerations during surgical planning for ulnar shortening osteotomy. a: Minimal acceptable distance between hole edge and osteotomy edge (set to be 1.5 mm). b: Drill hole diameter (set to be 2.5 mm). θ: Osteotomy slope angle (set to be 60°). x: Plate hole center distance (The hole center distance for small dynamic compression plate was 12 mm). y: Shortening distance (set to be 3 mm). z: Ulna thickness. r: Compression coefficient (1 mm for the dynamic compression plate). h: The distance between the third- and fourth-hole distance on the jig. 1. h = x + y + r. 2. If x-2*a-b + r-z*cotθ < 0, then choose a 7-hole dynamic compression plate instead of 6-hole

The system's design includes a main jig with multiple parallel and crossed pin holes for optimized temporary fixation. The cutting slot is designed to be swappable to improve the structural strength of the main jig and ease of use. To accommodate different shortening distances and ulna thicknesses, a simple algorithm was developed for quick specification referencing and plate selection (Fig. 1).

In this study, the shortening distance was set to 3 mm. The plate hole center distance (x) was 12 mm. The distance between the 3rd and 4th holes on the cutting jig (h) was calculated to be 12 + 3 = 15 mm. The ulna thickness (z) was set to be 9.5 mm, and a 60-degree chamfer angle was applied. Other tools in the system included a K-wire guide to facilitate accurate drilling and a temporary fixator for holding the osteotomy in place while applying the plate.

**Fig. 2:**
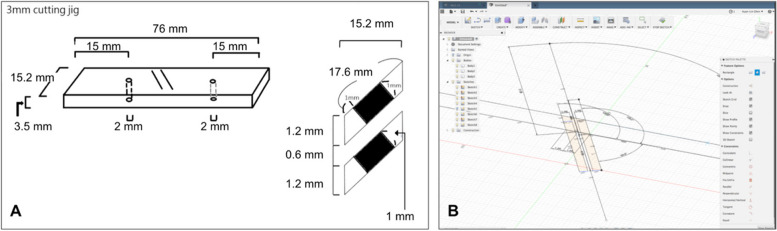
**A** The initial concept of the cutting guide system involving two cutting slots and two pin holes for temporary fixation. **B** The concept was further solidified using CAD software (Fusion360, AutoDesk, USA)

**Fig. 3:**
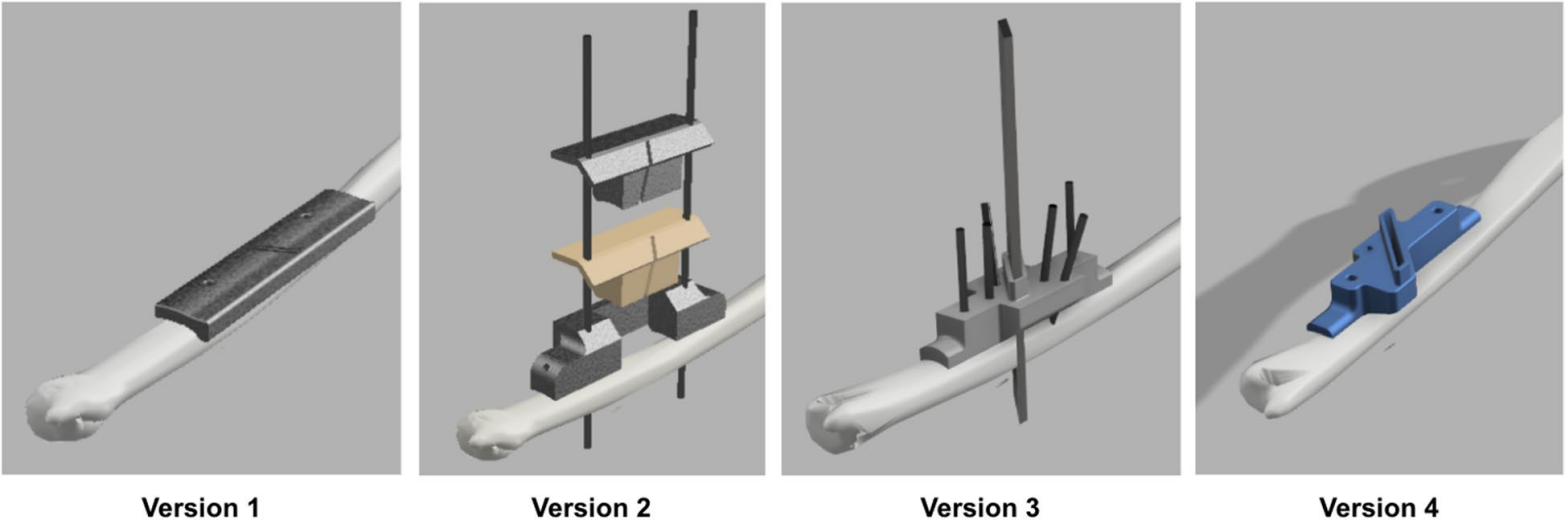
The different versions of the ulnar shortening system, from the first to the 4th

**Fig. 4:**
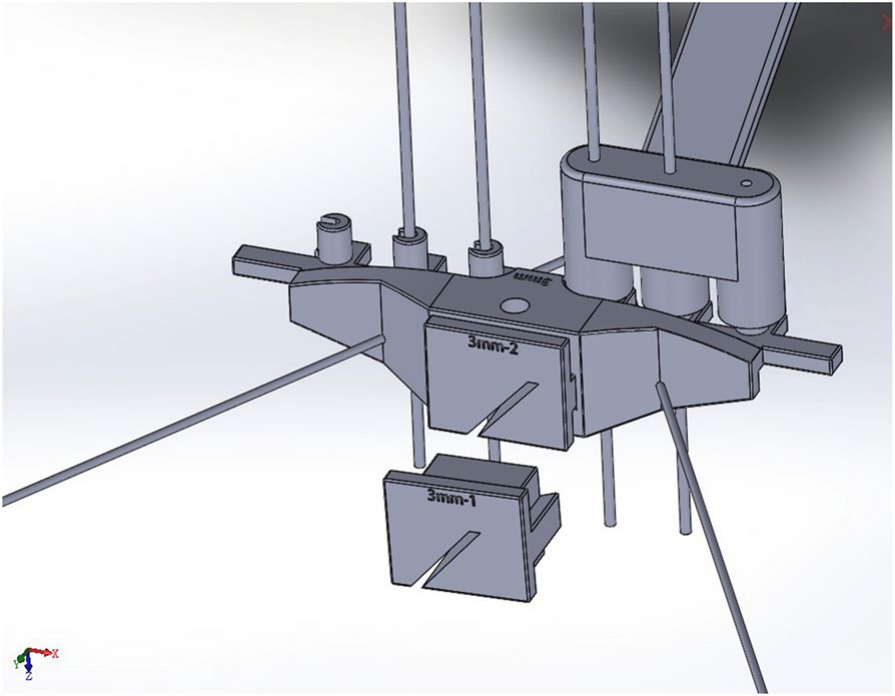
The CAD design of the 5th version of the 3.5 mm Dynamic Compression Plate Ulnar Shortening System

The initial concept (Fig. 2A) was first formalized through computer-assisted design (Fig. 2B). Many different versions were created during the development process (Fig. 3). Stereolithography (SLA)-manufactured prototypes were made for each version to demonstrate proof-of-concept and perform usability analysis. The final toolset was the fifth edition of this series of designs. After proper validation, the CAD files of the final version were refined using professional software (Solidworks 2018, USA) and then sent to an ISO 13485-certified metal additive manufacturing facility (Factory of Intelligent Additive Manufacturing Medical Devices, Industrial Technology Research Institute, Taiwan) (Fig. 4).

**Fig. 5:**
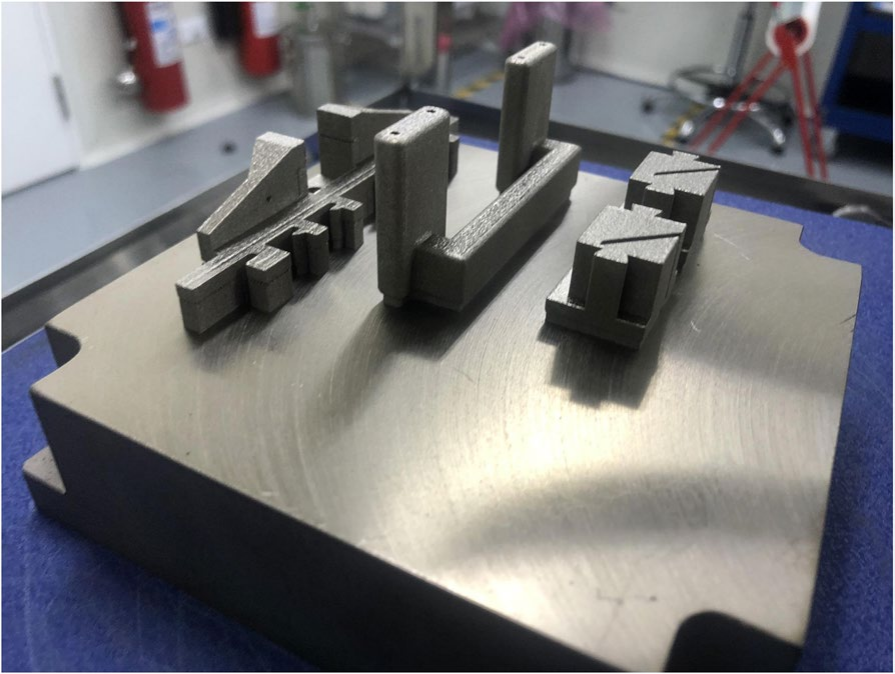
The parts on the metal powder bed with all powder removed

**Fig. 6:**
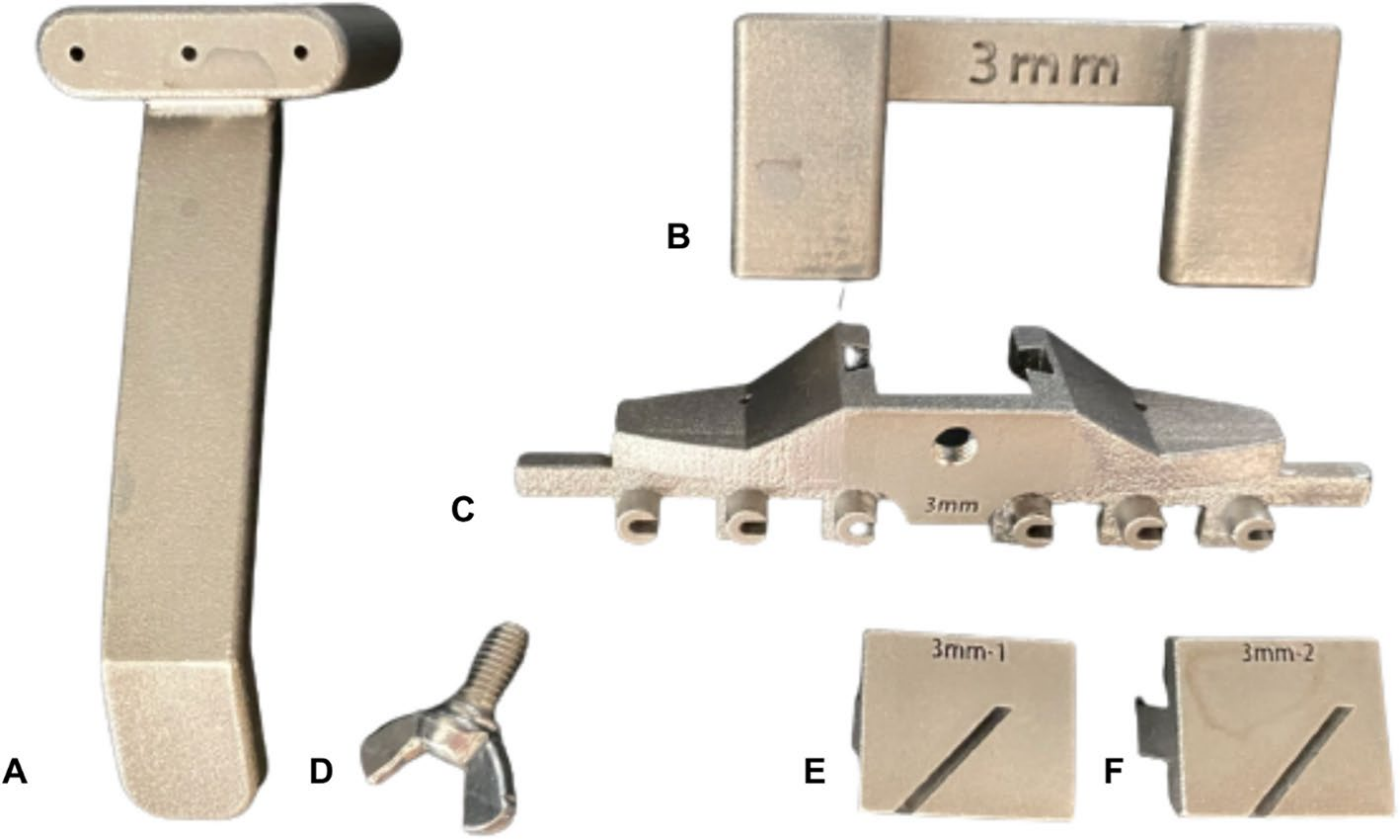
The additive manufactured 316 stainless steel 3.5 mm DCP ulnar shortening system after sandblasting **A** wire guide **B** temporary fixator **C** main jig **D** cutting block locking screw **E** cutting block-1 **F** cutting block-2

**Fig. 7:**
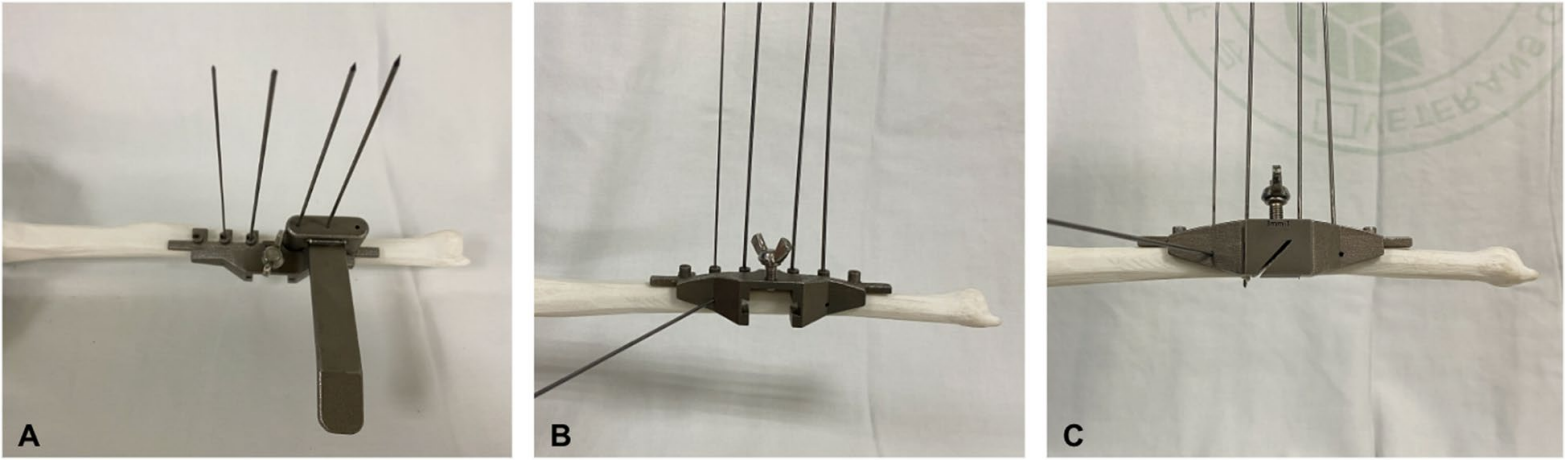
Assembly of the ulnar shortening guiding system. **A** Multiple K-wires inserted under the assistance of wire guide for to fix the main jig to the ulna. **B** Cross K-wire inserted through the main jig to reinforce jig stability **C** Cutting block-1 inserted to the main jig to start the first osteotomy

Before production, the CAD files underwent printability analysis and were placed in segmentation software for optimal print layout. Medical grade 316 stainless steel, regulated for medical devices, was selected. The instrument sets were then manufactured layer by layer using Selective Laser Sintering (SLS) technology on a metal powder bed (Fig. 5). The printed instrument sets were sandblasted for surface treatment. Subsequently, the tools were sent back to our institute for further testing. The entire process, from sending the finalized CAD files to receiving the parts, took approximately two weeks. The name, function of each part, and their assembly are shown in Figs. 6 and 7.

### Testing the ulnar shortening system

To test the performance of the system among surgeons with different levels of experience, we included two surgeons in the study. The first surgeon was a fellowship-trained hand and wrist surgeon. The second surgeon was a senior resident with a general understanding of the steps for free-hand ulnar shortening. The free-hand technique was illustrated in our previous publication [9]. Prior to testing, neither subject had experience using the ulnar shortening system described in this study. A step-by-step demonstration of how to use the system was given to each surgeon. Additionally, they were both provided with ulnar sawbones for practice before the actual testing.

**Fig. 8:**
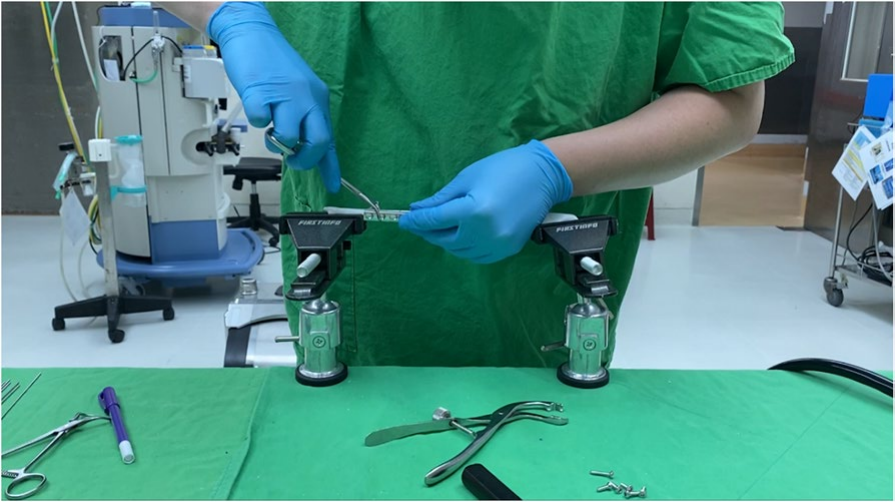
The setting during testing

Each surgeon performed three free-hand ulnar shortening osteotomies, followed by three instrument-assisted ulnar shortening osteotomies. The testing scheme is shown in Fig. 8. The recorded metrics included the time to complete the osteotomy, the time to complete implant fixation, chip thickness, shortening length, maximum residual gap, and deviation angle of the osteotomy.

### Operative procedure

#### Free-hand technique

The sawbone was fixed with two table clamps. An oblique line at a 60° angle along the ulnar shaft was marked 8 cm from the tip of the ulna styloid. A six-hole 3.5 mm dynamic compression plate was placed on the dorsal surface of the ulna and aligned along its long axis. The plate was held in place with reduction clamps, positioning the oblique mark in the middle of the plate. The third screw hole (from proximal to distal) was drilled at its center, while the fourth screw hole was drilled near the distal margin of the hole to facilitate compression. The plate was then removed.

The osteotomy was performed along the marked line using a microsagittal oscillating saw (HALL, USA), ensuring the cut was as parallel as possible. The bone chip from the cut was preserved and measured. The plate was then placed back on the bone and fixed with 3.5 mm screws in the predrilled third and fourth holes. After minor adjustments of the plate and screws for optimal reduction, the remaining screw holes were drilled and tightened with 3.5 mm cortical screws of the appropriate length.

####  3.5 mm DCP USO system

The sawbone was fixed with two table clamps. The main jig was placed on the dorsal surface of the ulna and aligned along its long axis. The main jig was then secured with reduction clamps. For temporary fixation, 1.6 mm K-wires were drilled into the holes on the main jig. Cutting block-1 was placed and fastened by tightening the cutting block screw. The first osteotomy was performed using a sagittal oscillating saw (HALL, USA) through the slot of cutting block-1.

**Fig. 9:**
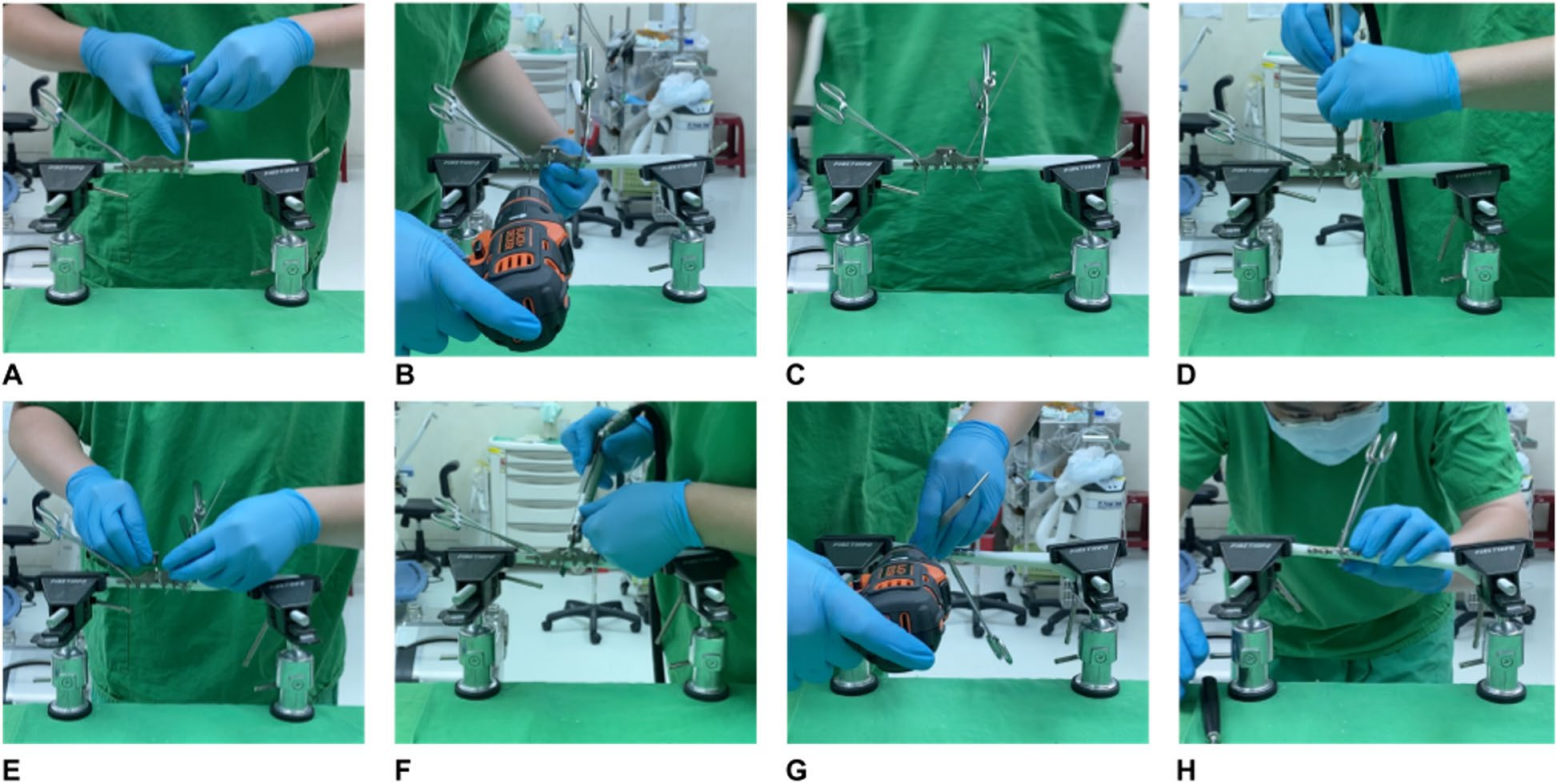
Critical steps of the instrument assisted USO with 3.5 mm DCP. **A** Main jig applied and secured with two reduction clamps. **B** 1.6 mm K-wires were placed for better temporary fixation. **C** Cutting block-1 placed and fastened. **D** Osteotomy under the guidance of cutting block-1. **E** Cutting block-1 replaced with cutting-block 2. **F** Osteotomy under the guidance of cutting block-2. **G** Main jig removed the pre-drilled holes were enlarged by 2.5 mm drill bit. The 6-hole 3.5 mm dynamic compression plate were fixated with six cortical screws. **H** Final plate position and reduction quality were confirmed

Next, cutting block-1 was removed and replaced with cutting block-2. After securing the cutting block, the second cut was completed using the microsagittal oscillating saw. The bone chip between the cuts was preserved and measured. A six-hole 3.5 mm dynamic compression plate was then placed through the K-wires. The osteotomy was held and reduced by passing the first, second, fifth, and sixth holes through the temporary fixator. The main jig was removed using the specifically designed open hole mechanism. After minor adjustments of the plate and temporary fixator for optimal reduction, the third and fourth K-wires were removed. Drilling and tapping were performed through the previous K-wire holes. Once optimal reduction was confirmed, all remaining K-wires were removed and replaced with screws for final fixation (Fig. 9).

### Measurements

Quantitative data, including surgical times, were collected and measured by a technician. The time to osteotomy was recorded from the start of the procedure to the completion of the osteotomy. The total procedure time was calculated from the start of the surgery to the tightening of the last screw. The chip diameter was measured as the thickest diameter of the removed bone chip. The shortening length was calculated by the difference in length between the tip of the ulna styloid and the olecranon process. The maximum residual gap was measured as the largest gap distance around the osteotomy site, between the proximal and distal segments.

To measure the deviation angle, the plate on each sawbone was removed, and the segments were reconnected with instant glue. The deviation angle was calculated by measuring the angle between the longitudinal axes of the proximal and distal segments.

### Statistical analysis

For the statistical analysis of the collected data, Student's t-test was performed to compare the differences between the osteotomy methods and level of experience. The analysis was conducted using SPSS software (version 25, IBM Corp., Armonk, NY, USA). The Student's t-test was used to assess the significance of differences in surgical times, chip diameter, shortening length, maximum residual gap, and deviation angle between the two methods. A *p*-value of less than 0.05 was considered statistically significant. The results were presented as mean ± standard deviation, providing insights into the precision and effectiveness of the instrument-assisted method compared to the traditional free-hand technique.

## Results

### Hand and wrist surgeon

The average time to complete an osteotomy was 468.7 ± 63.6 s. The time was significantly reduced to 260.0 ± 5 s using the USO instruments (*p* < 0.05). The total procedure time reported no statistically significant difference between free-hand USO and instrument-assisted USO. The chip diameter and total shortening length was larger in the free hand technique group. Despite the pre-operative shortening goal being 3 mm, the average shortening length using the free hand technique was 5 ± 0.1 mm. Both maximum residual gap and deviation angle reported no significant difference between the free-hand USO and instrument-assisted USO. Both the performance of the Hand and wrist surgeon and Senior resident doctor were reported in Table 1.

**Table 1:** The performance of data of Hand and wrist surgeon and Resident doctor

	Hand and wrist surgeon							Resident						
	Free hand	SD	CV	Instrument-assisted	SD	CV	*p*-value	Free hand	SD	CV	Instrument-assisted	SD	CV	*p*-value
Time to osteotomy (sec)	468.7	63.6	13.6%	260.0	5.0	1.9%	0.01*	344.7	128.1	37.2%	259.7	25.1	9.7%	0.15
Total procedure time (sec)	965.7	96.6	10.0%	948.0	114.8	12.1%	0.43	718.3	70.8	9.9%	1009.7	128.8	12.8%	0.02*
Chip diameter (mm)	2.6	0.4	13.9%	1.3	0.1	7.7%	0.01*	2.1	0.7	31.2%	1.2	0.1	4.9%	0.08
Shortening length	5.0	0.1	2.0%	3.2	0.2	5.4%	< 0.01*	3.6	0.7	17.9%	3.1	0.1	1.8%	0.17
Maximum residual gap	0.7	0.3	42.9%	0.8	0.6	78.1%	0.33	2.9	0.8	28.2%	0.4	0.4	81.0%	0.02*
Deviation angle (Degrees)	3.3	2.5	75.5%	0.7	1.2	173.2%	0.16	8.3	6.5	78.3%	0.3	0.6	173.2%	0.09

^*^p < 0.05: statistically significant; *SD* Standard deviation, *CV* Coefficient of variation

### Senior resident doctor

The average time to complete free hand USO was 344.7 ± 63.6 s while the time for instrument-assisted-USO was 259.7 ± 25.1 s. The procedure time for instrument-assisted USO was longer than free-hand-USO (1000.7, 718.3, *p* < 0.05). The chip diameter was thinner in the instrument-assisted-USO but without statistical significance. The shortening length showed no statistical difference among the two methods. The maximum residual gap was significantly larger in the free hand group (2.9 ± 0.8; 0.4 ± 0.4 mm, *p* = 0.02). The deviation angle was also greater in the free-hand group but did not achieve statistical significance.

### Inter-surgeon comparison

Inter-surgeon differences were also analyzed. Among free-hand USO, the procedure time was shorter for the resident doctor. The shortening length was greater for the hand and wrist specialist. In terms of quality of the osteotomy, both the maximum residual gap and deviation angle were significantly greater for the resident doctor. Among instrument-assisted-USOs, there were no significant differences between the hand and wrist specialist and resident doctor among all recorded parameters. All the inter-surgeon comparison results were shown in Table 2.

**Table 2:** Comparison between Hand and wrist surgeon and resident doctor in either Free-hand USO or Instrument-assisted USO

	Free hand					Instrument-assisted				
	Hand and wrist specialist	SD	Resident	SD	*p*-value	Hand and wrist specialist	SD	Resident	SD	*p*-value
Time to osteotomy (sec)	468.7	63.6	344.7	128.1	0.04*	260.0	5.0	259.7	25.1	0.49
Total procedure time (sec)	965.7	96.6	718.3	70.8	0.04*	948.0	114.8	1009.7	128.8	0.17
Chip diameter (mm)	2.6	0.4	2.1	0.7	0.17	1.3	0.1	1.2	0.1	0.29
Shortening length	5.0	0.1	3.6	0.7	0.03*	3.2	0.2	3.1	0.1	0.32
Maximum residual gap	0.7	0.3	2.9	0.8	0.03*	0.8	0.6	0.4	0.4	0.26
Deviation angle (Degrees)	3.3	2.5	8.3	6.5	0.08	0.7	1.2	0.3	0.6	0.21

^*^p < 0.05: statistically significant; *SD* Standard deviation

## Discussion

Ulnar shortening osteotomy is an established method in treating ulnar-sided wrist pain arouse from a positive ulnar variance. The basic concept involves unloading the ulnar-carpal joint. Traditionally, the osteotomy was done using free-hand technique. Technical notes have been published to improve the reproducibility of free-hand ulnar shortening osteotomy [9]. To optimize usability and precision, several commercially available USO systems have been developed [7, 8]. However, those systems are not widely available. Some patients could not afford the out-of-pocket payment for those systems. To tackle this challenge, our team developed a set of instruments for the 3.5 mm dynamic compression plate, which holds no out-of-pocket cost for under-resourced patients. This study showed that the developed system provides sufficient guidance for both hand surgeons and inexperienced resident doctors, producing consistently reliable results.

Non-union and malunion are two of the major complications of ulnar shortening procedure, leading to devastating consequences. It was reported that a residual gap of larger than 2 mm increases the incidence of non-union [10]. A reduction gap of less than 0.85 mm is essential to obtain union within 6 months. In the free-hand technique group done by resident doctor, the average maximum residual gap was 2.9 ± 0.8 mm, a value greater than the proposed threshold. In addition, the resident doctor produced larger average deviation angle (8.3 ± 6.5 degrees) with the free-hand technique despite no statistical significance. Large deviation angle indicates that the osteotomy site may not have good contact area after reduction and fixation, increasing the risk of non-union. On the contrary, the fellowship trained hand surgeon produced significantly better results, showing that free-hand technique is still technical demanding. The ulnar shortening system proposed in this study may help eliminate the steep learning curve of free-hand ulnar shortening osteotomy. Multiple features were designed to optimize effectiveness and mitigate pitfalls during the procedure. For making a precise shortening, the cutting jig must be fixated firmly to the ulna. To achieve this, multiple cross pin holes were made around the main jig. In addition, for precise shortening, saw blade thickness was considered. Because the blade measures 1.2 mm, if the cutting block was designed in one piece, the central septum dividing the two osteotomies would be too thin to resist the vibration from the saw. As a result, a swappable design was applied. The proximal and distal osteotomy were done in a serial fashion, using cutting block one and two. In addition to creating a precise osteotomy, making an accurate implant fixation without rotational and angulation deformity is essential. Predrilled technique was adopted for this purpose. The location and axis of the K-wires are designed to be identical to the final holes for screw fixation. The length and width of the main jig was designed to be the same as a 6-hole 3.5 mm dynamic compression plate to facilitate surgeons make easier estimation of final plate position. The adhering soft tissue can be cleaned optimally beforehand to avoid any impingement at the same time not too much for excessive periosteum damage. The temporary fixator was made for facilitating reduction and stabilizing the osteotomy before final screw placement.

Among the sawbones received instrument-assisted USO, unlike free-hand technique, there was no significant difference in the average maximum residual gap and deviation angle between the surgeons of different level of experience. With the assistance of the instrument set, there is a tendency to standardize the osteotomy and fixation, regardless of the surgeon's level of experience. Whether or not the instrument is used, there was no significant difference in the quality of osteotomy (maximum residual gap and deviation angle) for the hand and wrist specialist. The quality was both good and consistent. On the other hand, for the resident doctor, the quality of the osteotomy and fixation was significantly improved with the use of the instrument set. This finding was consistent with published literature, reporting that instrument-assisted-USO had a smaller osteotomy gap, shorter time to union and time to consolidation. In our study, the developed USO instrument set may flatten learning curve and improve consistency for USO, which is especially valuable for inexperienced surgeon.

The maturity of metal additive manufacturing technology enables surgeons to design patient-centered surgical instruments, which are not necessarily aligned with the business interests. Traditionally, orthopedic surgical instruments were mostly manufactured by CNC (Computer-neumeric control) machining, forging, and casting. The development of those manufacturing process for each component requires highly skillful technicians and thus are expensive. The need for substantial capital investment creates huge barrier to make products of limited market potential but may greatly benefit a small patient segment or underserved populations. In general, product development consists several steps, including concept development, sketching and computer-aided design, prototyping and reiterations, manufacturing process refinement. In this study, the total cost of bringing the idea to a final instrument set were significantly reduced through two major strategies. Firstly, after concept development, the sketch and the creation of computer-aided-design files were completed by a single orthopedic surgeon with an engineering degree. According to published data, the average hourly payment of an upper tier medical device design engineer was approximately 50–60 USD [11]. Calculated with the hours the surgeon spent on design and following iteration (> 120 h), the cost only for CAD design would be at least 6,000 USD. Secondly, with metal additive manufacturing technology, the production cost was significantly reduced. According to our record, if the instrument set were to be manufactured by CNC machining, the cost would be around 4,000 to 6,500 USD while the cost from metal additive manufacturing was 600 USD. Because the cost per unit may greatly reduce after the production line has been established, CNC manufacturing is more suitable for mass production.

Beyond production cost, additive manufacturing allows surgeon to embrace more design freedom in terms of geometry. The technical challenge of CNC machining and other traditional manufacturing technologies are making objects of complex geometries. The principle of CNC machining is by subtracting unwanted materials on a metal block by a computer-navigated burr until the remaining material meets the specification of the designed part [12]. Although multi-axial CNC machines have been developed, many complex geometries, especially deep, angled forms are still difficult to be manufactured this way [13]. For example, producing the main body (Fig. 6C) using traditional CNC machining was considered technically challenging. The design then had to be refined for manufacturability, incurring additional time and expertise costs. Instead of taking out material to form the final geometry as CNC machining, metal additive manufacturing creates geometry layer by layer through sintering metal powder point by point. Therefore, additive manufacturing allows the production of complex structures such as lattices [10]. For material used, multiple medical grade metals are available, including 316 stainless steel, Ti-6Al-4 V and Cobalt-chrome. Stainless steel was used for the instrument set for better stiffness. The turnover time, from CAD file to final component was 14 days in this study. In this study, because the instrument only requires a limited volume, additive manufacturing became the optimal method for final part production.

The process of surgeon-initiated instrument development depicted in this study is particularly valuable to creating patient-focused devices with expected small market share. Healthcare cost continue to rise and make up a substantial portion of family expenditure, especially in the US [14]. In the healthcare system where the study was conducted, with National Health Insurance, the out-of-pocket cost for patients was significantly more affordable than many other parts of the world. For an ulnar shortening osteotomy surgery, the out of pocket pay for the procedure itself was around 30 USD, which is affordable to most patients who need it [15]. However, specific USO systems were not reimbursed by the NHI, the out of pocket pay for those devices was around 2,200 USD, which was almost a hundred times more than the procedure [16]. Many patients thus were reluctant to use those systems. Furthermore, less experienced surgeons became reluctant to perform ulnar shortening osteotomy on those who were indicated because of the technical demand and potential risks coming from free-hand USO. The USO system developed in this study may make ulnar shortening osteotomy more widely available.

There are several limitations of this study. First, the test subjects were limited. More surgeons can be involved to produce a more convincing result. Secondly, our study lacked in vivo data. Further in vivo testing is warranted to understand the feasibility and performance of the system. Lastly, ISO-certified medical metal additive manufacturing facility was not widely available all around the world. The production cost may vary among different locations. However, partnerships for medical instrument original-equipment manufacturing were already in practice for decades. International collaborations should provide wide potentials.

## Conclusion

The ulnar shortening osteotomy system developed for the 3.5 mm dynamic compression plate in this study appears to be a safe and reliable tool for both experienced specialists and inexperienced surgeons to perform USO precisely and consistently. Additive manufacturing enables surgeons to develop surgical instruments that may optimize patient outcomes within a small budget. The process, from concept development, sketching, and creating CAD files to prototyping and iterations, and finally producing workable, certified instruments through additive manufacturing, can be translated to other surgical scenarios.

## Data Availability

All data are available upon request.

## Abbreviations

CAD: Computer-aided design
CNC: Computer-neumeric control
ISO-certified: International organization for standardization (ISO)-certified
SLA: Stereolithography
USD: United-States dollar
USO: Ulna shortening osteotomy
